# Developing a new endograft for the treatment of juxtarenal aortic aneurysms: definition and experimentation

**DOI:** 10.6061/clinics/2015(12)11

**Published:** 2015-12

## CLINICS 2015;70(6):435-40

In the article **Developing a new endograft for the treatment of juxtarenal aortic aneurysm: definition and experimentation** published in **CLINICS 2015;70(6),** on pages 435 and 440, **where it reads**:

“Lanzaiotti”

**it should read:**

“Lanziotti”

## CLINICS 2015;70(8):569-76

In the article **Glutamine treatment attenuates hyperglycemia-induced mitochondrial stress and apoptosis in umbilical vein endothelial cells** published in **CLINICS 2015;70(8),** on page 569, **where it reads**:

“Sher Zaman Safi,^I,*^ Kalaivani Batumalaie,^I^ Marzida Mansor,^II^ Karuthan Chinna,^III^ Syam Mohan,^IV^ Hamed Karimian,^IV^ Rajes Qvist,^I^ Muhammad Aqeel Ashraf,^V^ Garcie Ong Siok Yan^II^”

**and**

Safi SZ, Batumalaie K, Mansor M, Chinna K, Mohan S, Karimian H, et al. Glutamine treatment attenuates hyperglycemia-induced mitochondrial stress and apoptosis in umbilical vein endothelial cells. Clinics. 2015;70(8):569-576

**it should read:**

“Sher Zaman Safi,^I,*^ Kalaivani Batumalaie,^I^ Marzida Mansor,^II^ Karuthan Chinna,^III^ Hamed Karimian,^IV^ Rajes Qvist,^I^ Muhammad Aqeel Ashraf,^V^ Syam Mohan,^VI^ Garcie Ong Siok Yan^II^”

**and**

Safi SZ, Batumalaie K, Mansor M, Chinna K, Karimian H, Qvist R, et al. Glutamine treatment attenuates hyperglycemia-induced mitochondrial stress and apoptosis in umbilical vein endothelial cells. Clinics. 2015;70(8):569-576

**In the same article,** include the affiliation:

^VI^Medical Research Center, Jazan University, 11420, Jazan, Saudi Arabia.

